# Prediction of Early and Long-Term Hospital Readmission in Patients with Severe Obesity: A Retrospective Cohort Study

**DOI:** 10.3390/nu15163648

**Published:** 2023-08-20

**Authors:** Fabio Bioletto, Andrea Evangelista, Giovannino Ciccone, Amelia Brunani, Valentina Ponzo, Enrica Migliore, Eva Pagano, Isabella Comazzi, Fabio Dario Merlo, Farnaz Rahimi, Ezio Ghigo, Simona Bo

**Affiliations:** 1 Department of Medical Sciences, University of Turin, 10126 Turin, Italy; fabio.bioletto@unito.it (F.B.); valentina.ponzo@unito.it (V.P.); isabella.comazzi@unito.it (I.C.); ezio.ghigo@unito.it (E.G.); 2 Unit of Clinical Epidemiology, CPO, Città della Salute e della Scienza Hospital, 10126 Turin, Italy; andrea.evangelista@cpo.it (A.E.); gianni.ciccone@cpo.it (G.C.); enrica.migliore@cpo.it (E.M.); eva.pagano@cpo.it (E.P.); 3 Rehabilitation Medicine Unit, IRCCS Istituto Auxologico Italiano Piancavallo, 28824 Oggebbio, Italy; brunani@auxologico.it; 4 Dietetic Unit, Città della Salute e della Scienza Hospital, 10126 Turin, Italy; fdmerlo@gmail.com (F.D.M.); frahimi@cittadellasalute.to.it (F.R.)

**Keywords:** obesity, hospitalization, hospital readmission, predictive model

## Abstract

Adults with obesity have a higher risk of hospitalization and high hospitalization-related healthcare costs. However, a predictive model for the risk of readmission in patients with severe obesity is lacking. We conducted a retrospective cohort study enrolling all patients admitted for severe obesity (BMI ≥ 40 kg/m^2^) between 2009 and 2018 to the Istituto Auxologico Italiano in Piancavallo. For each patient, all subsequent hospitalizations were identified from the regional database by a deterministic record-linkage procedure. A total of 1136 patients were enrolled and followed up for a median of 5.7 years (IQR: 3.1–8.2). The predictive factors associated with hospital readmission were age (HR = 1.02, 95%CI: 1.01–1.03, *p* < 0.001), BMI (HR = 1.02, 95%CI: 1.01–1.03, *p* = 0.001), smoking habit (HR = 1.17, 95%CI: 0.99–1.38, *p* = 0.060), serum creatinine (HR = 1.22, 95%CI: 1.04–1.44, *p* = 0.016), diabetes (HR = 1.17, 95%CI: 1.00–1.36, *p* = 0.045), and number of admissions in the previous two years (HR = 1.15, 95%CI: 1.07–1.23, *p* < 0.001). BMI lost its predictive role when restricting the analysis to readmissions within 90 days. BMI and diabetes lost their predictive roles when further restricting the analysis to readmissions within 30 days. In conclusion, in this study, we identified predictive variables associated with early and long-term hospital readmission in patients with severe obesity. Whether addressing modifiable risk factors could improve the outcome remains to be established.

## 1. Introduction

Obesity is a chronic disease associated with a significant increase in morbidity and mortality [1,2,3]. Compared to the general population, patients with obesity are characterized by an increased risk of hypertension, diabetes mellitus, dyslipidemia, cardiovascular diseases, cancer, and several other health-related problems [1,4,5,6,7,8].

The prevalence of obesity is increasing globally [9,10], and it represents a major contributor to poor health in most countries [9,10,11]. This determines an ever-increasing burden on healthcare systems, with significant economic and social costs determined by obesity itself and by its associated complications [12,13,14]. Patients with obesity consume more healthcare resources compared with normal-weight individuals [12,13,14,15,16]. A remarkable part of these costs is determined by hospitalizations and inpatient care; indeed, adults with obesity are at increased risk of hospitalization compared to the general population [17,18,19,20], with high hospitalization-related healthcare costs [19,21,22,23].

A specific issue is represented by the risk of repeated hospital admissions. Overall, it has been estimated that approximately 13% of inpatients in the United States are responsible for utilizing over half of all hospital resources through repeated admissions [24,25]. During the last few decades, hospital readmission rates have been the subject of intense study in health economics [26,27,28], with the objectives of evaluating their causes, estimating their preventability, and assessing their validity as a measure of quality of care [24]. Most hospital readmissions occur shortly after a patient is discharged, with a subsequent decrease over time; approximately one-third occur within a month of discharge, half within 90 days, and 80% within a year [25,29,30]. In addition to being the most frequent, hospital readmissions occurring early after discharge have been commonly reported as the most predictable and preventable ones [31,32]. The percentage of preventable readmissions varies widely among studies, with estimates ranging from 9% to 48% [24]. This wide range is not surprising, given the high variability between different inpatient settings in terms of demographic, social, and disease-related characteristics [33,34,35].

Several predictive models have been proposed to predict the likelihood of hospital readmission in various clinical settings, such as acute myocardial infarction [36], heart failure [37,38,39], diabetes mellitus [40,41], chronic obstructive pulmonary disease [42], sepsis [43,44], among others. These models have been developed by including demographic and clinical variables that might influence readmission risk.

To the best of our knowledge, a predictive model for the risk of readmission in patients with severe obesity is still lacking. This study aimed to evaluate the predictors of hospital readmission in a selected cohort of patients hospitalized for the management of severe obesity; this evaluation was performed at different time points, i.e., within 30 days from previous hospital discharge, within 90 days from previous hospital discharge, and without time restrictions.

## 2. Materials and Methods

### 2.1. Patient Selection

All the patients residing in Piedmont (North-Western Italy) and admitted for severe obesity between 1 January 2009 and 31 December 2018 to the Istituto Auxologico Italiano (IAI) in Piancavallo were considered for inclusion. The IAI is a specialized tertiary care center for the treatment of severe obesity, accredited by the European Association for the Study of Obesity (EASO) as a Center for Obesity Management.

Admission to the IAI is reserved for the clinical management of patients with obesity with body mass index (BMI) ≥ 40 kg/m^2^, or with BMI 35–40 kg/m^2^ in the presence of obesity-related comorbidities, provided there is no concurrent disease that reduces life expectancy in the short term. During the admission at the IAI, a multidisciplinary program (nutritional, physical, and psychological intervention) is applied for about one month, according to patient needs.

To the scope of this study, the inclusion was restricted to patients with BMI ≥ 40 kg/m^2^. The only exclusion criterion was having undergone bariatric surgery before index admission. The study was approved by the Ethics Committee of the IAI (approval code #2019_10_22_01, approved on 22 October 2019) and was in accordance with the principles of the Declaration of Helsinki. Data was processed anonymously by working with encrypted personal identification codes. Due to organizational reasons and the high risk of introducing a selection bias in the study, consent to data processing could not be obtained from all patients.

### 2.2. Data Collection

The following data were collected from the medical records of the index admission at the IAI: age, sex, level of education, smoking habit, alcohol consumption, weight, height, BMI, waist circumference, ongoing medications, and fasting blood concentrations of creatinine and glucose.

All measurements were performed at the IAI by standardized procedures. Body weight (kg) was measured to the nearest 0.1 kg using a mechanical column scale (Scale-Tronix, Wheaton, IL, USA). Body height (cm) was measured to the nearest 0.5 cm using a stadiometer (Scale-Tronix, Wheaton, IL, USA). Waist circumference (cm) was measured with non-elastic tape at the level of the umbilicus. Biochemical analyses were performed in the local laboratory using standard methods, on samples collected in the morning after an overnight fast. Diabetes mellitus (type 2) was defined if a patient was receiving any anti-hyperglycemic treatment or had a fasting glucose level ≥126 mg/dL.

The hospital discharge records (HDRs) of the index admission were used to calculate, for each patient, the coded version of the Charlson Comorbidity Index, based on the International Classification of Diseases, ninth revision, and Clinical Modification (ICD9-CM) codes. In compliance with the definitions adopted by the Charlson index, cardiovascular disease was defined as the presence of congestive heart failure, peripheral vascular disease, prior myocardial infarction, or prior stroke/transient ischemic attack (TIA). End-stage kidney disease was defined as creatinine >3 mg/dL or ongoing dialytic treatment.

Furthermore, all HDRs from any hospital in Italy, between 2 years before the index admission until 31 December 2019, were identified from the regional database by a deterministic record-linkage procedure through the unique anonymous identifier code. Hospital admissions for the purpose of performing bariatric surgery were identified according to the presence of a main diagnosis of morbid obesity (ICD9-CM 278.01) and one of the following procedures: Roux-en-Y gastric bypass (ICD9-CM codes 44.31, 44.38), sleeve gastrectomy (ICD9-CM codes 43.89, 44.99), and adjustable gastric banding (ICD9-CM code 44.95); in the case of generic codes, the full HDR was checked to confirm the correct assignment. Information on patients’ deaths until 31 December 2019 was collected through a record linkage with the registry of the residents in the Piedmont Region by means of the unique anonymous code.

### 2.3. Statistical Analysis

Continuous data were summarized using mean and standard deviation (SD), unless otherwise specified. Categorical data were summarized using absolute frequencies and percent values.

The prediction of hospital admission was evaluated through three different Andersen–Gill Cox models for multiple-readmission-per-subject data [45]. The first and the second model assessed hospital readmission within 30 and 90 days from the previous discharge; in these two models, the observation times were censored at these specified time points. The third model assessed hospital readmission without any time constraint. Bariatric surgery and death were treated with censoring in all models. All collected variables were considered potential predictors and evaluated at univariate analysis. Afterward, they were assessed for inclusion in a multivariable model by a stepwise backward selection, using the Akaike Information Criterion (AIC) as the stopping rule [46]. The number of hospital admissions within the previous two years was treated as a time-dependent variable. The predictive performance of the model over time was evaluated through a time-varying area under the receiver operating characteristic curve (AUROC) for multiple-readmission-per-subject data.

A cut-off of 0.05 was adopted for the definition of statistical significance. Statistical analysis was performed using STATA 17 (StataCorp, College Station, TX, USA) and R 4.0.3 (R Foundation for Statistical Computing, Vienna, Austria).

## 3. Results

### 3.1. General Characteristics of the Study Population

After the application of all inclusion and exclusion criteria, a total of 1136 patients were enrolled. The mean age at the time of enrolment was 46.8 ± 10.8 years, and 62.3% of the patients were females. According to the inclusion criteria, all patients had a BMI ≥ 40 kg/m^2^, with a mean value of 47.4 ± 6.9 kg/m^2^. The other main clinical characteristics of the patients at the time of enrolment are reported in Table 1. The median follow-up period was 5.7 years (IQR: 3.1–8.2). During this time, a total of 2066 hospitalization occurred, with a rate of 0.34 hospitalizations per person-year. Overall, 158 patients (13.9%) underwent bariatric surgery after a median time of 2.9 years (IQR: 2.1–5.2). Patient death was registered in 71 (6.3%) cases, after a median time of 3.8 years (IQR: 2.0–6.2).

### 3.2. Predictive Factors for Hospital Readmission within 30 Days

Of the 2066 hospital admissions during the observation period, 194 (9.4%) occurred within 30 days from the previous hospital discharge. All collected variables were considered potential predictors for this outcome (Table 2). After the application of a stepwise backward selection, using the AIC as the stopping rule, four variables were retained in the final model (Table 2); these variables were age (HR = 1.02 per year, 95%CI: 1.00–1.05, *p* = 0.024), smoking habit (HR = 1.36, 95%CI: 0.96–1.94, *p* = 0.086), serum creatinine (HR = 1.34 per unit, 95%CI: 1.12–1.60, *p* = 0.001), and number of admissions in the previous two years (HR = 1.23 per unit, 95%CI: 1.14–1.30, *p* < 0.001).

### 3.3. Predictive Factors for Hospital Readmission within 90 Days

Of the 2066 hospital admissions during the observation period, 444 (21.5%) occurred within 90 days from the previous hospital discharge. All collected variables were considered potential predictors for this outcome (Table 3). After the application of a stepwise backward selection, using the AIC as the stopping rule, five variables were retained in the final model (Table 3); these variables were age (HR = 1.01 per year, 95%CI: 1.00–1.02, *p* = 0.055), smoking habit (HR = 1.27, 95%CI: 0.98–1.63, *p* = 0.066), serum creatinine (HR = 1.30 per unit, 95%CI: 1.11–1.55, *p* = 0.002), type 2 diabetes mellitus (HR = 1.24, 95%CI: 1.00–1.55, *p* = 0.050), and number of admissions in the previous two years (HR = 1.20 per unit, 95%CI: 1.16–1.25, *p* < 0.001).

### 3.4. Predictive Factors for Any Hospital Readmission

All collected variables were considered as potential predictors for hospital readmission also after broadening the analysis without restrictions related to the time from previous discharge (Table 4). After the application of a stepwise backward selection, using the AIC as the stopping rule, six variables were retained in the final model (Table 4); these variables were age (HR = 1.02 per year, 95%CI: 1.01–1.03, *p* < 0.001), BMI (HR = 1.02 per unit, 95%CI: 1.01–1.03, *p* = 0.001), smoking habit (HR = 1.17, 95%CI: 0.99–1.38, *p* = 0.060), serum creatinine (HR = 1.22 per unit, 95%CI: 1.04–1.44, *p* = 0.016), type 2 diabetes mellitus (HR = 1.17, 95%CI: 1.00–1.36, *p* = 0.045), and number of admissions in the previous two years (HR = 1.15 per unit, 95%CI: 1.07–1.23, *p* < 0.001).

### 3.5. Evaluation of Model Performance

**Figure 1:**
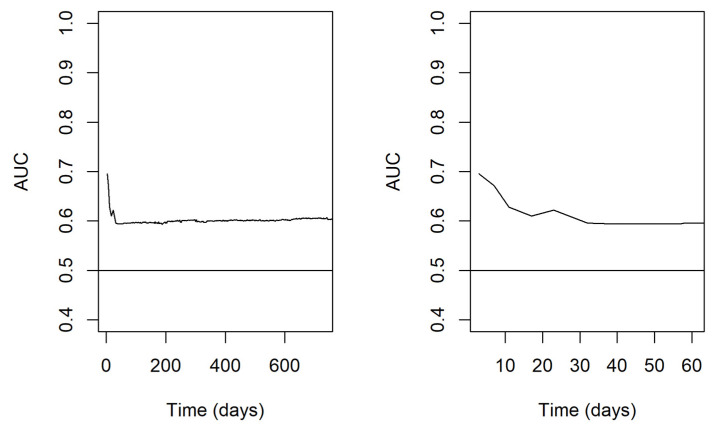
Time-varying AUROC for multiple-readmission-per-subject data. In the left panel, the results are reported on a wider time scale of 2 years; in the right panel, a narrower highlight on the first 2 months is proposed. Abbreviations: AUROC—area under receiver operating characteristic curve; AUC—area under curve.

The predictive performance of the model over time was evaluated through a time-varying AUROC for multiple-readmission-per-subject data (Figure 1). This evaluation showed a better predictive power of the model in the very early period after discharge, with an area under curve (AUC) starting from approximately 0.7 and then declining to 0.6 over the first 30 days. Thereafter, the predictive power of the model remained roughly stable at 0.6 up to 2 years.

## 4. Discussion

Our retrospective study examined the rates and the predictive factors associated with early and long-term hospital readmission in a large sample of adults with severe obesity.

Patients with obesity are at increased risk of hospitalization compared to the general population [17,18,19,20]. This fact points out the fragility of this patient cohort, and represents a relevant healthcare issue for several reasons; from the patient’s perspective, hospitalization can be a disruptive and stressful experience, with a significant impact on their mental and physical well-being, independence, and social support network [47,48]; from the healthcare perspective, repeated hospitalizations are related to higher healthcare costs [19,21,22,23]. Moreover, unplanned hospital readmission has been proposed as a performance indicator of health care quality [49,50], although this point is up for debate. Several authors reported a significant association between early hospital readmission and low-quality inpatient care [34], but others have noted that high readmission rates might be linked to lower mortality and may indicate a high level of severity of illness and intensity of care [35]. Understanding these complex dynamics is crucial for developing effective strategies to improve outcomes and reduce the burden on both patients and healthcare systems.

Various predictive models have been suggested for estimating readmission rates across different clinical settings [36,37,38,39,40,41,42,43,44]. Nevertheless, our findings are difficult to compare with the existing literature because, as far as we know, no previous research has been focused specifically on the prediction of hospital readmission in a selected cohort of patients hospitalized for the management of severe obesity. This fact emphasizes the need for tailored insights to address the distinctive challenges and dynamics within this specific patient population.

In this study, we focused our attention on identifying predictors of early and long-term readmission in our cohort. In multivariable analyses, we found a considerable overlap among variables predicting 30-day, 90-day, and long-term readmission, with age, smoking, number of admissions in the previous two years, and serum creatinine being predictive factors in all three models.

The role of age and smoking as risk factors for hospitalizations is well established. Older adults are more likely to experience acute medical conditions and injuries that require hospital admission [36,40,43]. Smoking is strongly associated with increased morbidity due to its causal role in a range of medical conditions such as cardiovascular disease, respiratory illness, and cancer [51,52,53]; people who smoke are more likely to require hospitalization for these conditions, and quitting smoking can reduce the risk of hospitalization and improve overall health outcomes [54].

The importance of previous admissions as a predictor for future re-hospitalizations is not surprising, as they represent a summary indicator of patient recent adverse health events, and they may be considered as a proxy that evaluates his/her fragility [55]. Interestingly, this has already been demonstrated also in the specific setting of obesity, in which temporal data extracted from historical patient hospitalization records in a one-year timeframe significantly increased the predictive performance for 30-day hospital readmission of patients with morbid obesity [56]. These findings emphasize the utility of incorporating longitudinal patient history into predictive models, as it provides a more holistic view of patient frailty, with a better estimation of readmission risk.

In addition to these factors, the serum creatinine value has also been detected as a predictive factor for hospital readmission in our cohort. This result is consistent with other studies in which serum creatinine was found to be a predictor of future readmissions in various contexts [57,58,59]. Serum creatinine value is commonly used for the measurement of kidney function [60], but in patients with obesity, it might be also strongly influenced by non-renal factors [61]. Being a product of muscle catabolism [62], its values may have been underestimated in our cohort, given that sarcopenic obesity is a quite common condition in patients with severe obesity [63]. The effect of sarcopenia on serum creatinine levels may have masked possible kidney damage, and this could explain why we found creatinine as a predictor of readmission despite values that, in our cohort, were mostly within or close to the normal range.

In addition to the predictors listed above, we found that type 2 diabetes mellitus is independently associated with 90-day and long-term readmissions. This finding is not unexpected, considering that multiple hospitalizations are common among diabetic patients [64]. Literature data reported that diabetes was associated with an increased risk of readmission in patients hospitalized for cardiac surgery [65], heart failure [66], acute myocardial infarction [67], stroke [68], or liver disease [69].

Interestingly, in the multivariable analysis, BMI acted as a predictor only in the long term, while it had a neutral effect on 30-day and 90-day readmission. Thus, it seems that, in our cohort, short-term readmissions were mostly related to comorbidities rather than the severity of obesity per se. Furthermore, this might also be a part of the so called “obesity paradox”, which refers to the phenomenon by which obesity may also act as a protective factor in some specific circumstances, including hospital readmissions [70,71]. A possible explanation for this “obesity paradox” might be that obesity may protect against malnutrition, which often occurs during hospitalization [67] and is typically associated with higher readmission rates [72]. These findings highlight the complex interplay between obesity, comorbidities, and hospital readmissions, suggesting the need for further research to fully understand the underlying mechanisms.

With respect to model performance, we found that readmissions occurring within 30 days from previous discharge were the most predictable, as AUC values were higher within 30 days and then decrease for longer periods. This finding confirms what has been demonstrated in other clinical settings, in which readmissions occurring within 30 days from previous discharge appeared to be the most predictable and preventable ones [31,32]. In absolute terms, the predictive performance of the model was only moderate, starting from approximately 0.7, declining to 0.6 over the first 30 days, and thereafter remaining roughly stable up to 2 years. However, this result is comparable to the performance of other predictive models for hospital readmission developed in different clinical settings [37,38,40,44]. This is not surprising, given the multitude of conceptual factors that may influence readmission risk and that are outside of the hospital’s purview. These findings underscore the complex nature of predicting readmission outcomes and highlight the need for a holistic approach that considers various factors to enhance the accuracy of predictive models.

The main strengths of this study were the large sample size, as well as the length and completeness of the follow-up. Another strength was the centralized anthropometric assessment measured at a single center by trained professionals, with baseline information available on several established risk factors known to be associated with subsequent morbidity, which allowed us to account for possible confounders.

Our study had limitations. First, there might be residual or unmeasured confounding variables that could have impacted the results. Second, the anthropometric and biochemical assessments were performed only at the time of enrolment; this might contribute to limiting the discriminative ability of the models as the time since discharge from IAI increases. Third, concerns might arise with respect to the generalizability of our findings; in fact, our cohort consisted of patients admitted to a specialized tertiary care center for the treatment of severe obesity and might not be fully representative of the overall population of patients with severe obesity. However, since the residential nutritional rehabilitation program provided by the IAI does not include active treatments for obesity (e.g., bariatric surgery) or its related complications, its influence on the subsequent patterns of hospitalization is expected to be minimal. Moreover, all subsequent hospital admissions after the first discharge from the IAI took place in other general hospitals due to a wide variety of reasons and might be assumed to roughly reflect a “real world” scenario.

## 5. Conclusions

In this study, we identified and evaluated the predictive factors associated with early and long-term hospital readmission in patients with severe obesity. Overall, the predictive performance of the obtained multivariable model is only moderate, likely due to the multitude of unmeasurable confounding factors that may influence readmission risk. Nevertheless, these estimates shed light on conceptual aspects that should be considered when quantifying the risk of hospital readmission in a patient with severe obesity. Whether the management of modifiable risk factors could improve the outcome and reduce the patient’s readmission rate remains unclear and needs to be established.

## Figures and Tables

**Table 1:** Characteristics of the population at the time of enrolment. Abbreviations: COPD—chronic obstructive pulmonary disease; n—number; SD—standard deviation.

Parameter	Value in the Overall Cohort (*n* = 1136)
Age (years), mean ± SD	46.8 ± 10.8
Sex	
Male, *n* (%)	428 (37.7)
Female, *n* (%)	708 (62.3)
Education level	
High school or higher, *n* (%)	443 (39.0)
Intermediate school, *n* (%)	572 (50.4)
Elementary school or less, *n* (%)	121 (10.6)
Weight (kg), mean ± SD	128.0 ± 24.0
Height (cm), mean ± SD	163.6 ± 10.2
BMI (kg/m^2^), mean ± SD	47.4 ± 6.9
Waist (cm), mean ± SD	131.8 ± 14.7
Smoking habit	
No, *n* (%)	835 (73.5)
Yes, *n* (%)	301 (26.5)
Alcohol consumption	
No, *n* (%)	686 (60.4)
Yes, *n* (%)	450 (39.6)
Charlson Index	
0, *n* (%)	621 (54.7)
1, *n* (%)	388 (34.1)
≥2, *n* (%)	127 (11.2)
Cardiovascular disease	
No, *n* (%)	1125 (99.0)
Yes, *n* (%)	11 (1.0)
COPD	
No, *n* (%)	1073 (94.5)
Yes, *n* (%)	63 (5.5)
Creatinine (mg/dL), mean ± SD	0.79 ± 0.39
End-stage kidney disease	
No, *n* (%)	1119 (98.5)
Yes, *n* (%)	17 (1.5)
Type 2 diabetes mellitus	
No, *n* (%)	817 (71.9)
Yes, *n* (%)	319 (28.1)
N. of hospital admissions in the previous 2 years, mean (range)	0.7 (0–22)

**Table 2:** Predictive factors for hospital readmission within 30 days. Abbreviations: BMI—body mass index; CI—confidence interval; COPD—chronic obstructive pulmonary disease; HR—hazard ratio; n—number; ref—reference.

Parameter	Univariate Analysis			Multivariable Analysis		
	HR	95%CI	*p*-Value	HR	95%CI	*p*-Value
Age (per year)	1.03	0.99–1.06	0.106	1.02	1.00–1.05	0.024
Sex						
Male	1 (ref)					
Female	0.84	0.53–1.34	0.467			
Education level						
High school or higher	1 (ref)					
Intermediate school	1.31	0.81–2.13	0.276			
Elementary school or less	1.44	0.85–2.44	0.170			
Weight (per unit, kg)	1.00	0.99–1.01	0.464			
Height (per unit, cm)	0.99	0.98–1.01	0.451			
BMI (per unit, kg/m^2^)	0.99	0.96–1.02	0.479			
Waist (per unit, cm)	1.00	0.99–1.02	0.726			
Smoking habit						
No	1 (ref)			1 (ref)		
Yes	1.14	0.74–1.74	0.555	1.36	0.96–1.94	0.086
Alcohol consumption						
No	1 (ref)					
Yes	0.88	0.56–1.37	0.558			
Charlson Index						
0	1 (ref)					
1	0.90	0.57–1.45	0.676			
≥2	1.28	0.71–2.31	0.418			
Cardiovascular disease						
No	1 (ref)					
Yes	1.67	0.63–4.46	0.306			
COPD						
No	1 (ref)					
Yes	1.26	0.68–2.35	0.466			
Creatinine (per unit, mg/dL)	1.53	1.12–2.10	0.008	1.34	1.12–1.60	0.001
End-stage kidney disease						
No	1 (ref)					
Yes	1.91	0.98–3.72	0.056			
Type 2 diabetes mellitus						
No	1 (ref)					
Yes	1.20	0.79–1.80	0.393			
N. of hospital admissions in the previous 2 years (per unit)	1.23	1.17–1.29	<0.001	1.23	1.16–1.30	<0.001

**Table 3:** Predictive factors for hospital readmission within 90 days. Abbreviations: BMI—body mass index; CI—confidence interval; COPD—chronic obstructive pulmonary disease; HR—hazard ratio; n—number; ref—reference.

Parameter	Univariate Analysis			Multivariable Analysis		
	HR	95%CI	*p*-Value	HR	95%CI	*p*-Value
Age (per year)	1.02	1.00–1.03	0.092	1.01	1.00–1.02	0.055
Sex						
Male	1 (ref)					
Female	0.93	0.69–1.26	0.637			
Education level						
High school or higher	1 (ref)					
Intermediate school	1.24	0.91–1.70	0.178			
Elementary school or less	1.45	0.97–2.17	0.069			
Weight (per unit, kg)	1.00	0.99–1.01	0.989			
Height (per unit, cm)	1.00	0.99–1.01	0.926			
BMI (per unit, kg/m^2^)	1.00	0.98–1.02	0.856			
Waist (per unit, cm)	1.01	1.00–1.02	0.188			
Smoking habit						
No	1 (ref)			1 (ref)		
Yes	1.13	0.84–1.51	0.422	1.27	0.98–1.63	0.066
Alcohol consumption						
No	1 (ref)					
Yes	0.96	0.72–1.29	0.801			
Charlson Index						
0	1 (ref)					
1	0.87	0.63–1.19	0.378			
≥2	1.41	0.98–2.03	0.064			
Cardiovascular disease						
No	1 (ref)					
Yes	1.01	0.54–1.89	0.981			
COPD						
No	1 (ref)					
Yes	1.28	0.89–1.83	0.175			
Creatinine (per unit, mg/dL)	1.37	1.09–1.72	0.008	1.30	1.11–1.55	0.002
End-stage kidney disease						
No	1 (ref)					
Yes	2.01	1.06–3.80	0.032			
Type 2 diabetes mellitus						
No	1 (ref)			1 (ref)		
Yes	1.25	0.94–1.68	0.128	1.24	1.00–1.55	0.050
N. of hospital admissions in the previous 2 years (per unit)	1.20	1.16–1.25	<0.001	1.20	1.16–1.25	<0.001

**Table 4:** Predictive factors for any hospital readmission. Abbreviations: BMI—body mass index; CI—confidence interval; COPD—chronic obstructive pulmonary disease; HR—hazard ratio; n—number; ref—reference.

Parameter	Univariate Analysis			Multivariable Analysis		
	HR	95%CI	*p*-Value	HR	95%CI	*p*-Value
Age (per year)	1.02	1.01–1.03	<0.001	1.02	1.01–1.03	<0.001
Sex						
Male	1 (ref)					
Female	0.94	0.80–1.10	0.427			
Education level						
High school or higher	1 (ref)					
Intermediate school	1.03	0.87–1.21	0.743			
Elementary school or less	1.27	0.99–1.62	0.056			
Weight (per unit, kg)	1.00	1.00–1.01	0.118			
Height (per unit, cm)	1.00	0.99–1.01	0.840			
BMI (per unit, kg/m^2^)	1.01	1.00–1.02	0.057	1.02	1.01–1.03	0.001
Waist (per unit, cm)	1.01	1.00–1.01	0.047			
Smoking habit						
No	1 (ref)			1 (ref)		
Yes	1.03	0.87–1.23	0.736	1.17	0.99–1.38	0.060
Alcohol consumption						
No	1 (ref)					
Yes	1.02	0.87–1.20	0.790			
Charlson Index						
0	1 (ref)					
1	1.02	0.86–1.20	0.833			
≥2	1.29	1.00–1.66	0.048			
Cardiovascular disease						
No	1 (ref)					
Yes	1.07	0.63–1.82	0.807			
COPD						
No	1 (ref)					
Yes	1.29	0.99–1.68	0.060			
Creatinine (per unit, mg/dL)	1.30	1.08–1.56	0.005	1.22	1.04–1.44	0.016
End-stage kidney disease						
No	1 (ref)					
Yes	2.30	1.39–3.81	0.001			
Type 2 diabetes mellitus						
No	1 (ref)			1 (ref)		
Yes	1.24	1.05–1.46	0.010	1.17	1.00–1.36	0.045
N. of hospital admissions in the previous 2 years (per unit)	1.15	1.07–1.24	<0.001	1.15	1.07–1.23	<0.001

## Data Availability

The data presented in this study are available upon request from the corresponding authors.
